# Matrix Metalloproteinase Mediated Type I Collagen Degradation is an Independent Predictor of Increased Risk of Acute Myocardial Infarction in Postmenopausal Women

**DOI:** 10.1038/s41598-018-23458-4

**Published:** 2018-03-29

**Authors:** Ditte Marie Bertelsen, Jesper Skov Neergaard, Cecilie Liv Bager, Signe Holm Nielsen, Niels Henry Secher, Jesper Hastrup Svendsen, Asger Reinstrup Bihlet, Jeppe Ragnar Andersen, Morten Asser Karsdal, Claus Christiansen, Henning Bay Nielsen

**Affiliations:** 1 ProScion, Herlev, Denmark; 2 Department of Anaesthesia, Rigshospitalet, University of Copenhagen, Copenhagen, Denmark; 3 Biomarkers and Research, Nordic Bioscience, Herlev, Denmark; 4 Department of Biotechnology and Biomedicine, Technical University of Denmark, Lyngby, Denmark; 5 Department of Cardiology, The Heart Centre, Rigshospitalet, University of Copenhagen, Copenhagen, Denmark; 6 Clinical Development, Nordic Bioscience, Herlev, Denmark

## Abstract

Acute myocardial infarction (AMI) is often underdiagnosed in women. It is therefore of interest to identify biomarkers that indicate increased risk of AMI and thereby help clinicians to have additional focus on the difficult AMI diagnosis. Type I Collagen, a component of the cardiac extracellular matrix, is cleaved by matrix metalloproteinases (MMPs) generating the neo-epitope C1M. We investigated the association between serum-C1M and AMI and evaluated whether C1M is a prognostic marker for outcome following AMI. This study is based on The Prospective Epidemiological Risk Factor (PERF) Study including postmenopausal women. 316 out of 5,450 women developed AMI within the follow-up period (14 years, median). A multivariate Cox analysis assessed association between serum-C1M and AMI, and re-infaction or death subsequent to AMI. The risk of AMI increased by 18% (p = 0.03) when serum-C1M was doubled and women in the highest quartile had a 33% increased risk compared to those in the low quartiles (p = 0.025). Serum-C1M was, however not related to reinfarction or death subsequent to AMI. In this study C1M was be an independent risk factor for AMI. Measuring MMP degraded type I collagen could be useful for prediction of increased risk of AMI if replicated in other cohorts.

## Introduction

Coronary heart disease (CHD) affects mainly men but is also a leading cause of mortality in women^1–3^. The incidence of AMI rises after menopause and the mean age of women with first-time AMI is higher than for men (72 vs. 65 years)^4^. Mehta *et al*. reported in a Statement from American Heart Association that despite improvement in treatment modalities, women are more likely to die from acute myocardial infarction (AMI) than men^1^. For women AMI seems not to be studied adequately, misdiagnosed, and therefore left untreated. Additionally, evidence for prognostic factors to predict adverse outcome after AMI in women is sparse^5^. There is a need for novel biomarkers that assess the risk and prognosis of AMI in women to improve survival^5^.

Atherosclerosis is an inflammatory process characterized by accumulation of plaques and plaque calcification. Nodules erotion and rupture are complications leading to AMI and could be indicated by the epitope C1M. Plaque rupture is the most common cause of AMI by triggering the coagulation cascade whereby the risk of thrombosis is accelerated. A ruptured plaque has a necrotic core with an overlying thin fibrous cap infiltrated by inflammatory cells^4,6^ and expression of macrophage-released matrix metalloproteinases (MMPs) affects plaque stability^7^. MMP-induced proteolytic cleavage of collagen may weaken the fibrous cap and increase the risk of plaque rupture, which will trigger the thrombotic cascade leading to AMI^4,8–11^ and detection of plaque instability is of interest.

Cavusoglu *et al*.^4^ report elevated MMP-3 levels in plasma to be associated with five-year-risk of AMI in men. In postmenopausal women, Dragsbaek *et al*.^12^ demonstrated the MMP-2, −9, and −13 generated type I collagen degradation fragment (C1M) to be associated with cardiovascular mortality. This study evaluated whether C1M is linked to AMI in postmenopausal women. C1M data presented by Dragsbaek *et al*.^12^ were further analysed to evaluate (*i*) association between serum-C1M and AMI in postmenopausal women and (*ii*) baseline serum-C1M as a biomarker for short-term prognosis following AMI.

## Materials and Methods

### Study population

This evaluation is based on the cohort “The Prospective Epidemiological Risk Factor (PERF) Study” which, in a prospective observational design includes Danish postmenopausal women to investigate age-related diseases. A detailed description of the cohort have previously been published^13,14^. The PERF study was conducted in accordance with Good Clinical Practice and approved by the Danish National Committee on Health Research Ethics (KA99070gm) with informed consent obtained from all subjects. Women who had participated in clinical randomized placebo-controlled studies or had been screened for the studies at the Centre for Clinical and Basic Research in Denmark were invited to participate in PERF. In 1999, 8,875 postmenopausal women were invited to attend a baseline examination. 5,855 women, aged 49–89 years consented to attend baseline examination for the PERF study (between 1999–2001) with no other in- or exclusion criteria. For the present evaluation subjects without a record of AMI at baseline and with a complete dataset were selected for analysis. Thus, the study was conducted without a predefined sample size estimate.

### Baseline

Fasting blood samples were collected and serum stored at −80 °C. Participants completed a physical examination by non-blinded staff including height and weight. Information in regard to physical activity, smoking, alcohol consumption, and diabetes was obtained through a structured interview questionnaire. Serum analysis, retrieval of information from The National Danish Patient Registry and The National Danish Causes of Death Registry were blinded.

### Outcome Variables

In Denmark, all citizens are provided a unique personal identification number (CPR) that enables matching individuals to the Danish health registries. Data on AMI diagnosis, AMI date, cause and date of death were extracted from The National Danish Patient Registry and The National Danish Causes of Death Registry on 31/12/2014. Data from The National Danish Patient Registry and The National Danish Causes of Death Registry were matched with the PERF cohort. Diagnosis was classified in accordance with the International Classification of Diseases: Diagnosis code I21-I21.09 (AMI), tenth revision (ICD-10) or diagnosis code 410–410.9 (AMI) for the eighth revision (ICD-8). Both AMI as diagnosis and cause of death during the follow-up were included until 31/12/2014. The secondary outcomes included subsequent AMI incidents. A secondary study outcome (class A) was death (regardless of registered cause) or re-infarction within 28 days after an AMI according to the WHO definition^15^. Any outcome not involving death or re-infarction within 28 days was categorized as a class B outcome. For the purpose of defining classes A and B, the follow-up started on the day of the first AMI and ended at death within 28 days thereafter, re-infarction within 28 days from the AMI, or on 31/12/2014, whichever came first. Additional episodes of re-infarction or death related to a recurrent infarct were not included in the analysis.

### Matrix Metalloproteinase Mediated Type I Collagen Degradation

A matrix metalloproteinase (MMP)-linked immunosorbent assay (ELISA), which detects serum fragments of type I collagen generated by MMP-2, −9, and −13 (termed C1M) was developed and validation with assay variability is published^16^. While other biomarkers of type I collagen fragments exist and used as measure of bone turnover^17–19^, the MMP generated fragment is destroyed by cathepsin-K indicating that this collagen fragment is not derived from bone turnover. The C1M fragment was analysed prior to previous studies and found to be stable after storage of serum at −80 °C for 12 years. In short, C1M stability was at the same time confirmed by seven consecutive freeze-thaw cycles with no significant variation in detected levels. Three-year stability studies were performed at the same time to validate C1M detection by measuring the same sample with a one-year interval^12^. The association between C1M levels and age at baseline was tested with a Pearson-correlation and found not to be significant (r = 0.01, p = 0.45). Finally, the C1M levels are comparable to those found in other cohorts. No blood samples and thus no measurements of C1M from the time of infarction were available.

### Statistical Analysis

Data are presented as median with interquartile range (IQR) if not otherwise specified. Analyses were performed in MedCalc® (version 14.8.1) and RStudio® (version 3.3.1) and a p-value <0.05 was considered significant. The study population was stratified in two groups: A group included participants who were found to develop AMI and a group who did not develop AMI during the follow-up. Baseline characteristics were compared using a Mann-Whitney test for numerical variables. Categorical variables were compared using a Chi-square test. The cohort was divided into quartiles based on serum-C1M levels: Q1: 21.2–31.1 ng/mL (n = 1,406), Q2: 31.4–39.5 ng/mL (n = 1,392), Q3: 39.6–56.0 ng/mL (n = 1,384), and Q4: 56.1–2240 ng/mL (n = 1,389). A Kaplan-Meier Survival Curve was applied to demonstrate incident events of AMI over time in relation to the four quartiles of C1M-levels. Multivariate Cox proportional hazard analysis assessed association between C1M levels and incident AMI. Event-free mortality was included as a competing risk by censoring. The follow-up time since baseline was the time scale. All further analyses were conducted with C1M-quartiles, Q1–3 vs. Q4, and log_2_-transformed serum-C1M levels. The Q1–3 vs. Q4 cut off was chosen based on results from the Kaplan-Meier Survival Curve. The analysis was adjusted for potential confounders including “Major Risk Factors and Coronary Heart Disease” (age, BMI, diabetes mellitus, hypertension, serum-cholesterol, tobacco smoking and physical inactivity) according to American Heart Association^20^ and alcohol consumption ≥7 units/week cf. recommendation by the Danish Health Authority in regard to elderly^21^. Age, BMI, serum-cholesterol and -C1M were included in the analysis as numerical variables. The categorical variables were (*i*) diabetes divided into no, insulin-dependent or noninsulin-dependent, (*ii*) hypertension categorized as presence of hypertension or no hypertension, blood pressure systolic >139 mmHg or diastolic >89 mmHg^22^, (*iii*) smoking classified as never, previous or current, (*iiii*) level of physical activity grouped in relation to engagement in activities other than daily living, and (*iiiii*) use of alcohol defined as consumption of more or less than 7 units per week (15 mL or 12 g of pure alcohol). The multivariate Cox analysis was likewise used to evaluate serum-C1M as a prognostic marker for AMI outcome within 28 days after the event. The proportional-hazard assumption was checked by interacting covariates over time within the model adhering to STROBE guidelines.

## Results

### Study Population

**Figure 1:**
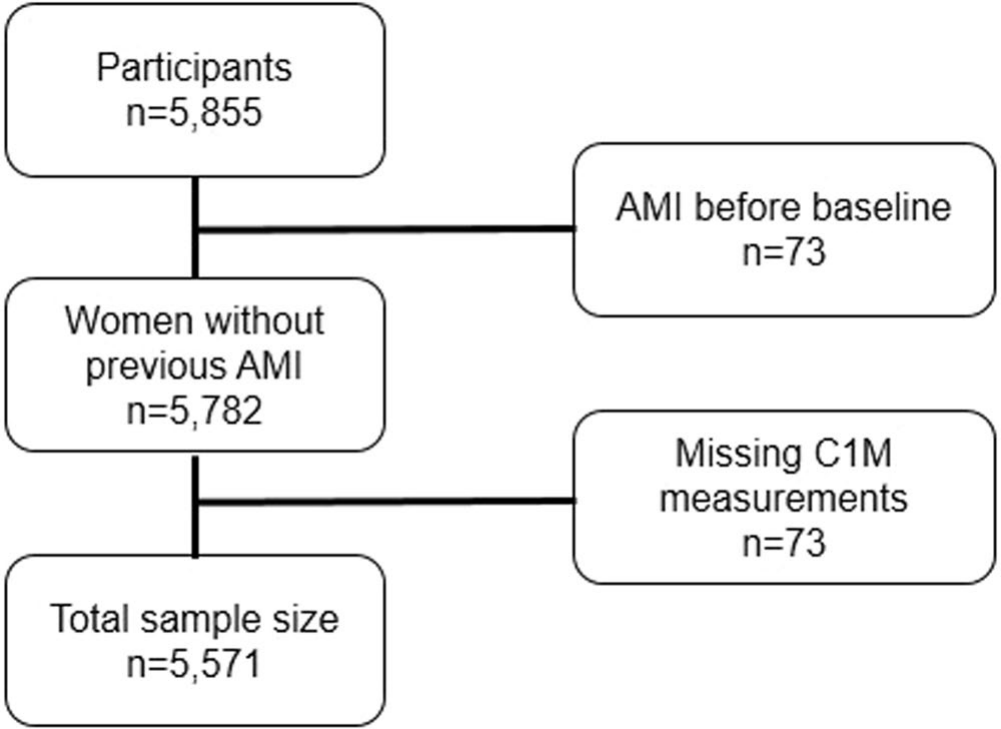
Flow-chart, study population.

5,855 participants were screened, but 73 subjects had a record of AMI before baseline and serum-C1M was missing for 211 women. Thus, the study population included 5,571 (Fig. 1) of whom 316 developed AMI during the follow-up. In 121 participants there were missing data (such as blood pressure, BMI, serum-cholesterol, diabetes) and the statistical analyses on a full data set involved 5,450 women with AMI occurring in 309 (5.7%) participants. In comparison, 18 (5.4%) events of AMI occurred in women who were excluded (n = 332) with no statistical difference between the two groups (p = 0.85). The median (IQR) follow-up time was 14.0 years (13.7–14.4) and time to AMI 6.5 years (2.9–10.5); for subjects in Q1 6.4 (3.2–10.9), Q2 7.3 (2.7–11.4), Q3 8.8 (3.6–10.8), and Q4 5.1 years (2.5–9.8).

### Baseline Characteristics

Table 1 summarizes baseline characteristics for the included participants. Their age ranged from 51 to 86 years (70.9 (65.5–75.5), median with IQR) and women with AMI were older. The population had a BMI of 25.7 (23.2–28.7) kg/m^2^ of which the AMI group had the highest value. Additionally, serum-cholesterol was 6.3 (5.6–7.0) mmol/L with higher levels in the AMI group. Likewise, in women with AMI, 7.1% presented with diabetes and 78.3% with hypertension compared to only 2.6% and 67%, respectively, in women without AMI. In addition, the prevalence of current smokers was higher in the AMI group whereas previous smokers dominated the non-AMI group. The proportion of participants with significant alcohol consumption was similar in the two groups, but the AMI group was less physically active.

**Table 1:** PERF cohort baseline characteristics: Selected variables cf. American Heart Associations “Major Risk Factor and CHD”. The variables are given as percentage or median (IQR).

Variable	Total (n = 5,450)	AMI (n = 309)	No-AMI (n = 5,141)	P-value AMI vs no-AMI
Age (years)	70.9 (65.5–75.5)	74 (69.1–78.2)	70.7 (65.3–75.3)	<0.001
BMI (kg/m^2^)	25.7 (23.2–28.7)	26.7 (23.9–29.3)	25.7 (23.2–28.6)	0.005
Diabetes				
Insulin-dependent	121 (2.2%)	16 (5.2%)	105 (2.0%)	<0.001
Noninsulin-dependent	34 (0.6%)	6 (2.0%)	28 (0.5%)	<0.001
Diastolic blood pressure (mmHg)	81 (74–90)	82 (75–91)	81 (74–90)	0.2823
Systolic blood pressure (mmHg)	148 (133–166)	155 (140–174)	148 (132–165)	<0.0001
Hypertension	3686 (67.6%)	242 (78.3%)	3444 (67%)	<0.001
Serum-cholesterol (mmol/L)	6.3 (5.6–7.0)	6.4 (5.8–7.1)	6.3 (5.6–7.0)	0.04
Smoking				
Previously	1660 (30.5%)	84 (27.2%)	1576 (30.7%)	0.009
Current	1204 (22.1%)	91 (29.4%)	1113 (21.6%)	0.0016
Alcohol (≥7 units/week)	1789 (32.8%)	86 (27.8%)	1703 (33.1%)	0.063
Physical inactivity	1673 (30.7%)	118 (38.2%)	1555 (30.2%)	0.004
Serum-C1M (ng/mL)	39.5 (31.3–56.0)	43.7 (32.3–66.0)	39.4 (31.2–50.7)	0.005
Serum-C1M				
Q1	1374 (25.2%)	66 (21.4%)	1308 (25.4%)	0.0086
Q2	1360 (25.0%)	71 (23.0%)	1289 (25.1%)	
Q3	1357 (24.9%)	70 (22.7%)	1287 (25.0%)	
Q4	1359 (24.9%)	102 (33.0%)	1257 (24.5%)	

### C1M and AMI

Serum-C1M was 39.5 (31.3–56.0) ng/mL and highest in subjects with incident AMI during the study (43.7 (32.3–66.0) vs. 39.4 (31.2–50.7); p = 0.005). Hence, participants with AMI demonstrated an 11.5% higher serum-C1M when data were evaluated unfiltered by a potential influence of risk factors.

**Figure 2:**
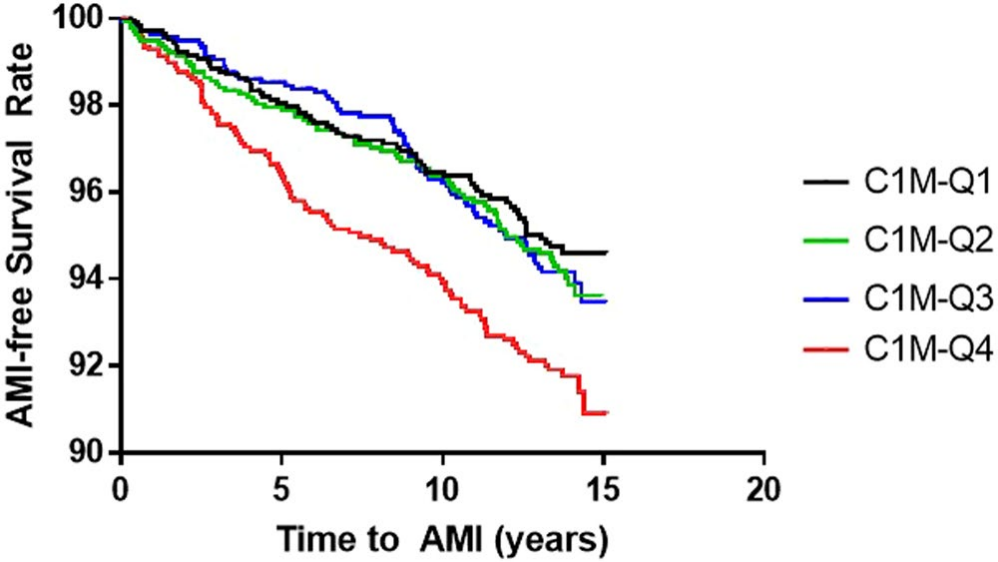
Kaplan-Meier Survival Curve of C1M quartiles. The lowest quartile, C1M-Q1, is illustrated in black, C1M-Q2 is illustrated in green, C1M-Q3 is illustrated in blue and the highest quartile of C1M-Q4 is illustrated in red. Cutoff C1M >56 ng/mL.

Figure 2 is the Kaplan-Meier Survival Curve, stratified in quartiles of C1M to illustrate the association between C1M and AMI. The plot shows a decrease in AMI-free survival rate for women with baseline serum-C1M levels in the upper quartile (Q4) compared to women with C1M levels in the lower quartiles (Q1–Q3) (p = 0.0055).

Based on the results from Fig. 2 and to adjust for possible confounders, a multivariate Cox proportional-hazard analysis was performed. C1M was analysed as a continuous variable (log_2_ transformed), divided into quartiles and using a binary outcome (Q4 vs. Q1–3) (Table 2). The independent contribution of high C1M levels to develop AMI with analysis adjusted for age, BMI, diabetes mellitus, hypertension, serum-cholesterol, tobacco smoking, physical inactivity, and alcohol consumption is evaluated. The log_2_-tranformed C1M analysis found that with a doubled given C1M-level, the hazard ratio for AMI increases by 18% (p = 0.03). For women with C1M-levels in the highest quartile the risk for AMI increased by 33% compared to C1M-levels in the quartile 1–3 (p = 0.025).

**Table 2:** Multivariate Cox proportional-hazard analysis for C1M-levels divided into quartiles associated with AMI. Corrected for age, body mass index, diabetes mellitus, hypertension, serum-cholesterol, tobacco smoking, physical inactivity, and alcohol consumption. *p < 0.05.

Variable	Hazard ratio	95% confidence interval	P-value
C1M-Q			
Q1 Q2 Q3 Q4 C1M-cutoff	Ref. 1.06 1.0 1.35 1.33	[0.76; 1.48] [0.71; 1.40] [0.98; 1.87] [1.04; 1.70]	0.75 0.98 0.068 0.025*
Log_2-_C1M	1.18	[1.02; 1.38]	0.03*

In Table 3, evaluation on class A vs. B outcomes in relation to C1M levels are presented with 117 (A) and 199 (B) women classified. The class A outcomes included reinfarction n = 54, recurrent AMI as cause of death n = 55, and death within 28 days after recurrent AMI (registered cause other than AMI) n = 8. With analysis adjusted for age, data failed to reach statistical significance illustrating that C1M was not able to predict prognosis within 28 days following an AMI.

**Table 3:** Multivariate Cox proportional-hazard analysis for baseline C1M as a prognostic marker for reinfarct or death within 28 days as a measure of short-term AMI outcome following the first AMI, regardless of when the first AMI occurred.

Variable	Hazard ratio	95% confidence interval	P-value
C1M-Q			
Q2 Q3 Q4 C1M-cutoff	1.33 0.78 1.33 1.29	[0.77; 2.30] [0.43; 1.43] [0.79; 2.24] [0.88; 1.89]	0.31 0.42 0.28 0.19
Log_2-_C1M	1.18	[0.79; 1.26]	0.97

## Discussion

This study is based on data from the PERF study including Danish postmenopausal women and assessed whether serum-C1M is associated to risk of AMI. C1M was evaluated for its potential to reflect an unstable atherosclerotic plaque and consequently whether elevated C1M levels is associated with risk of plaque rupture and thrombosis. A second aim was to evaluate whether C1M correlates to outcome within a short period subsequent to AMI.

The study demonstrates that a high C1M (>56 ng/mL), as a surrogate for MMP mediated type I collagen tissue remodelling^16^, is associated with risk of AMI in postmenopausal women, independent of overweight, diabetes, hypertension, hyperlipidaemia, smoking, alcohol consumption, and inactivity. Within a 14-year follow-up period, postmenopausal women with high C1M levels (>56 ng/mL) demonstrated a 33% higher risk for AMI compared to women with C1M levels in the lower quartiles. Thus, the data suggest C1M as a biomarker for increased risk of AMI in postmenopausal women. This study did, however not demonstrate C1M as a prognostic marker for outcome following the first AMI, when C1M is measured prior to first AMI.

Due to an ageing population and improved therapeutic options for cardiac diseases the prevalence of cardiovascular diseases is increasing and triggers morbidity, mortality, and increased health care costs. According to the WHO 2014 Global Status Report on non-communicable diseases, CVD is the leading cause of death. In 2012, approximately 17.5 million people died from CVD and of these 7.4 million (42.3%) died because of AMI. Thus, improved methods to assess the risk of AMI are warranted.

We measured MMP-degraded type I collagen because increased levels of C1M could reflect breakdown and destabilisation of the fibrous cap of an atherosclerotic plaque. Plaque instability and subsequent rupture leading to trombosis is thought to occur due to destruction of the fibrous cap, rich in type I collagen, mediated by macrophage-derived MMPs^8,23–25^. This study showed that C1M, reflecting MMP-mediated breakdown of collagen type I in the fibrous cap, is associated with AMI. This hypothesis is supported by Cavusoglu *et al*.^4^ in a study of 355 male veterans referred to diagnostic coronary angiography. They found plasma MMP-3 (at a pre-specified cut-off of 20.56 ng/mL) associated to 5-year risk of AMI. The risk of plaque rupture is difficult to detect^6^ with plaque stability investigated by Near-Infrared Spectroscopy, laser based imaging, and Polarization-Sensitive Tomography, but success has been limited^6,9,10,25^.

High levels of C1M were associated with increased risk of AMI in postmenopausal women. To our knowledge we are the first to investigate MMP-degraded collagen in relation to risk of AMI. However, other extracellular matrix proteins have been investigated in relation to AMI and mortality. The endothelium is topped with glycocalyx and syndecan-1 is a biomarker of glycocalyx degradation. Ostrowski *et al*.^26^ examined plasma syndecan-1 in 571 patients with ST-myocardial infarction treated by percutaneous coronary intervention and a high syndecan-1 level was independently correlated to 30-day cardiovascular- and all-cause mortality in two univariate analyses (p = 0.012 and p = 0.002). However, when adjusting for cardiovascular risk factors, the predictive value for mortality within 30 days became insignificant and syndecan-1 did not associate to reinfarction within that time frame. The multivariate Cox analysis on the prognostic value of C1M for re-infarction or death after AMI was also not significant may be because the blood samples were collected up to 15 years prior to the AMI.

Plasma-MMP-3, syndecan-1, and here serum-C1M demonstrate potential new biomarkers for identification of subjects with elevated risk of AMI, but C1M was not a prognostic marker for risk of death or re-infarction subsequent to AMI. Yet, Dragsbaek *et al*.^12^ found C1M associated to mortality and the level of C1M at the time of AMI may be related to outcome, e.g. mortality and could be determined immediately after AMI.

The strength of this paper includes a large and homogenous population without inclusion- or exclusion criteria and the participants are considered to be representative for Danish postmenopausal women. Additionally, the access to Danish patient registries is valuable; registry-linkage collected outcome information implies limited loss of data. Limitations are also identified. Autopsies are seldom performed when elderly Danes die even unexpectedly and some cases of AMI may not have been identified rendering a potential bias in the age composition of the population. Additionally, information about medication was not available and blood samples were collected up to 14.4 years prior to AMI. Thus, analysis of C1M association with short-term outcome following AMI would likely improve had C1M been obtained at that time. Thus, in this study C1M is a novel marker for long-term risk assessment. CRP measurements were unavailable for this cohort. Moreover, the potential link between cardiovascular- and connective tissue disease was not addressed and diagnostic criteria for AMI have changed during the follow-up^27^. Finally, Danish geography and organization of health care may not necessarily apply to other countries and as the data are restricted to postmenopausal Danish Caucasian women, the conclusions may not be generalized to the overall population.

In conclusion, in this study including postmenopausal women serum-C1M was associated to development of AMI and high levels seems to be an independent risk factor prior to AMI. External validation is warranted to confirm the results of this paper.
